# Anti-Obesity Effect of 6,8-Diprenylgenistein, an Isoflavonoid of *Cudrania tricuspidata* Fruits in High-Fat Diet-Induced Obese Mice

**DOI:** 10.3390/nu7125544

**Published:** 2015-12-15

**Authors:** Yang Hee Jo, Kyeong-Mi Choi, Qing Liu, Seon Beom Kim, Hyeong-Jin Ji, Myounghwan Kim, Sang-Kyung Shin, Seon-Gil Do, Eunju Shin, Gayoung Jung, Hwan-Soo Yoo, Bang Yeon Hwang, Mi Kyeong Lee

**Affiliations:** 1 College of Pharmacy, Chungbuk National University, Cheongju, Chungbuk 28644, Korea; qow0125@naver.com (Y.H.J.); mirine0101@hanmail.net (K.-M.C.); liuqing7115@hotmail.com (Q.L.); suntiger85@hanmail.net (S.B.K.); yoohs@chungbuk.ac.kr (H.-S.Y.); byhwang@chungbuk.ac.kr (B.Y.H.); 2 Laboratory Animal Research Center, Chungbuk National University, Cheongju, Chungbuk 28644, Korea; jihjin@chungbuk.ac.kr (H.-J.J.); mhkim3340@naver.com (M.K.); skshin@chungbuk.ac.kr (S.-K.S.); 3 Wellness R&D Center, Univera, Inc., Seoul 05014, Korea; sgildo@univera.com (S.-G.D.); Ejayshin@univera.com (E.S.); gayoung@univera.com (G.J.)

**Keywords:** 6,8-Diprenylgenistein (DPG), *Cudrania tricuspidata*, high-fat diet-induced obesity, lipid profile, AMPK

## Abstract

Obesity, which is characterized by excessive fat accumulation, is associated with several pathological disorders, including metabolic diseases. In this study, the anti-obesity effect of 6,8-diprenylgenistein (DPG), a major isoflavonoid of *Cudrania tricuspidata* fruits was investigated using high fat-diet (HFD)-induced obese mice at the doses of 10 and 30 mg/kg for six week. The body weight of the DPG-treated groups was significantly lower compared to the HFD-treated group. In addition, fat accumulation in epididymal adipose tissue and liver was dramatically decreased in the HFD + DPG groups. The food efficiency ratios of the HFD + DPG groups were also lower compared to the HFD group with the same food intake. Metabolic parameters that had increased in the HFD group were decreased in the HFD + DPG groups. Further studies demonstrate that DPG efficiently reduces lipogenic genes by regulation of transcription factors, such as peroxisome proliferator-activated receptor γ (PPARγ) and CCAAT/enhancer-binding protein α (C/EBPα), and hormones, such as leptin and adiponection. DPG also regulates acetyl-CoA carboxylase (ACC) and hydroxy-3-methylglutaryl coenzyme A reductase (HMGCR) by AMP-activated protein kinase (AMPK) activation. Taken together, DPG is beneficial for the regulation of obesity, especially resulting from high fat intake.

## 1. Introduction

The prevalence of obesity has increased continuously and became one of the major threats to global health due to the association with several pathological disorders, including diabetes, hypertension, atherosclerosis and cancer [1,2]. In the development of obesity, genetic and environmental factors are known to play important roles. In particular, a diet high in saturated fats is considered to be one of main contributors to obesity, particularly in the Western diet. Fat is digested into monoglycerides and fatty acids by lipase and absorbed fat is accumulated in adipose tissue through excessive adipocyte differentiation [3]. Adipocyte differentiation is an organized process regulated by various transcriptional factors depending on differentiation stages. CCAAT/enhancer-binding protein (C/EBP) β and δ are expressed in early stages of adipocyte differentiation. C/EBPs β and δ regulate the expression of peroxisome proliferator-activated receptor γ (PPARγ) and CCAAT/enhancer-binding protein α (C/EBPα), the most crucial transcriptional factors in adipogenesis [4]. Obesity is also closely related to the levels of leptin and adiponectin, adipose specific hormones. Leptin amount is proportionally correlated with obesity, while adiponection amount is inversely related to obesity [5]. Therefore, inhibition of adipogenesis by the regulation of transcription factors and hormones are one of the targets of anti-obesity strategies. AMPK is another regulator of cellular energy homeostasis. Phosphorylation by AMPK inactivates metabolic enzymes, such as acetyl CoA carboxylase (ACC) and hydroxy-3-methylglutaryl coenzyme A reductase (HMGCR), which results in fatty acid oxidation and reduced biosynthesis of fatty acid and cholesterol [6,7].

Phytochemicals are defined as the substances found in edible fruits and vegetables. Various phytochemicals are known to exhibit a potential for human health. In addition, favorable role of phytochemicals on the prevention and treatment of obesity have been reported. Epigallocatechin gallate of green tea is well known for its beneficial effect on obesity [8]. Xanthigen, a mixture of brown seaweed and pomegranate seed extract, showed anti-obesity activity in animal model [9]. Sulforaphane, an isothicyanate of broccoli and alkaloids of lotus leaves, showed beneficial effects on obesity [10,11].

Fruits are good sources of diverse phytochemicals. Especially, polyphenols, such as flavonoids, are considered as major functional components of fruit. They possess diverse biological activities (e.g., antioxidant, estrogenic, anti-inflammatory and anti-cancer activity [12,13,14]. As a result, the utility of this fruit as an ingredient in dietary supplements and functional foods ingredients is being actively investigated in many fields.

**Figure 1:**
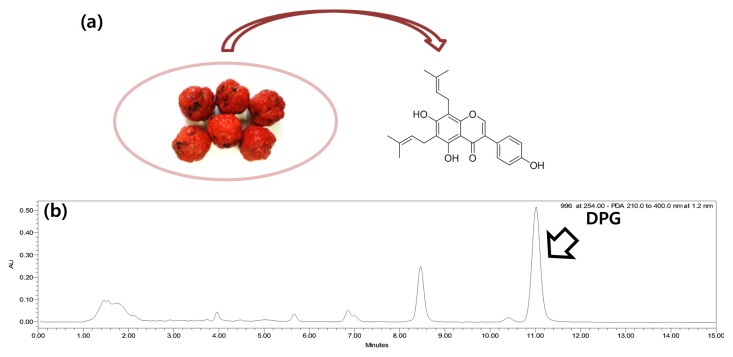
(**a**) Structure of 6,8-diprenylgenistein (DPG) from *C. tricuspidata* fruits; (**b**) High performance liquid chromatography (HPLC) chromatogram of the extract of *C. tricuspidata* fruits.

*Cudrania tricuspidata,* which belongs to the Moraceae family, is a thorny tree cultivated in East Asia including Korea. The fruits of *C. tricuspidata* are widely consumed as fresh fruits, jams, and processed products, such as wine and vinegar. They are known for its diverse biological activities such as antioxidant, anti-inflammatory and immunomodulatory activity [15,16,17]. We previously reported the pancreatic lipase inhibitory activity of *C. tricuspidata* fruits. 6,8-diprenylgenistein (DPG), a major isoflavonoid of *C. tricuspidata* fruits (Figure 1) was suggested as an active constituent [18]. Moreover, the inhibitory activity of DPG on diacylglycerol acyltransferase (DGAT), a key enzyme in triglyceride synthesis, has been reported [19]. Therefore, further studies have been attempted to elucidate anti-obesity effect in high fat diet (HFD)-induced animal model and its potential mechanisms, to verify the anti-obesity activity of DPG.

## 2. Experimental Section

### 2.1. Chemicals

Antibodies against peroxisome proliferator-activated receptor γ (PPARγ) and hydroxy-3-methylglutaryl coenzyme A reductase (HMGCR) were purchased from Santa Cruz Biotechnology, Inc. (Santa Cruz, CA, USA). Antibodies against CCAAT/enhancer-binding protein α (C/EBPα), adiponectin, phospho-AMP-activated protein kinase alpha (p-AMPKα), phospho-AMP-activated protein kinase β1 (p-AMPKβ), phospho-acetyl-CoA carboxylase (p-ACC), and β-actin were obtained from Cell Signaling Technology (Beverly, MA, USA). The antibody against leptin was obtained from GeneTex, Inc. (Irvine, CA, USA).

### 2.2. Preparation of DPG

DPG, a major isoflavonoid of *C. tricuspidata* fruits was isolated, as previously reported [18] (Figure 1a,b). The purity was higher than 95%, as measured HPLC analysis.

### 2.3. Animal Treatment

Male C57BL/6J mice (*n* = 60, 4 weeks old, 15% ± 10% g) were purchased from Central Lab. Animal Inc., Seoul, Korea. All mice were housed in a room with controlled temperature (21–23 °C), humidity (55%–60%), and lighting (12-h light/dark cycle) and given water *ad libitum*. After acclimation for 1 week, the mice were randomly divided into the following four groups (*n* = 15/group): ND (normal diet), HFD, HFD + DPG10 (10 mg DPG/kg mice) and HFD + DPG30 (30 mg DPG/kg mice). ND group was fed with a normal diet (4.3% fat of diet, 7% of kcal from fat). The HFD, HFD + DPG10 and HFD + DPG30 groups were fed HFD (24% fat including 0.5% cholesterol of diet, 45% of kcal from fat) (Table S1). DPG samples were prepared in distilled water (DW) at the concentrations of 10 mg/mL or 30 mg/mL. DPG samples were administered orally (10 mL/kg) to HFD + DPG groups, and DW (10 mL/kg) was administered orally to the ND and HFD groups for 6 weeks. At the end of experiment, the mice were sacrificed and their blood and organs were collected. The protocol for this study was approved by the Animal Care and Use Committee of Chungbuk National University (Approval No. CBNUA-644-13-01).

### 2.4. Serum Biochemical Parameters

All mice were fasted for 12 h before they were sacrificed. Blood was collected and serum was obtained by the centrifugation at 3500 g for 10 min at 4 °C. The content of triglyceride, total cholesterol, low-density lipoprotein (LDL) cholesterol, high-density lipoprotein (HDL) cholesterol, alanine transaminase (ALT) and aspartate transaminase (AST) were determined using Hitachi7080 analyzer.

### 2.5. Histopathology

Histological photograph of adipose tissue was analyzed based on the paraffin method using a light microscope. Epididymal adipose tissue was fixed with 10% neutral buffered formalin and embedded in paraffin block. Six μm sections were cut and mounted on glass slide. Paraffin was removed with xylen and alcohol. The sections were then stained with hematoxylin and eosin (H&E). After dehydration by alcohol, the photograph was taken with light microscope. The size of epididymal adipocyte was calculated by Image analysis system (IPKR-1003, Saramsoft Co., Ltd., Seoul, Korea). For the detection of lipid deposition in liver, liver section were prepared from frozen liver and stained with Oil Red O, as previously reported [20].

### 2.6. Western Blot Analysis

Epididymal adipose tissues were homogenized in lysis buffer containing 50 mM Tris-HCl (pH 7.4), 1% Triton X-100, 0.2% sodium deoxycholate, 0.2% sodium dodecylsulfate, 1 mM phenylmethylsulfonyl fluoride, and a protease inhibitor cocktail tablet. The homogenate was centrifuged at 18,300 g for 30 min at 4 °C, and the supernatant was collected. The total protein concentration of each lysate was measured using bicinchoninic acid (BCA) Protein Assay Reagent. Proteins in the lysates were electrophoretically separated in 7.5% to 15% SDS polyacrylamide gel and then transferred to polyvinylidene difluoride membranes. The membranes were blocked in 5% bovine serum albumin overnight at 4 °C and then incubated overnight at 4 °C with the following primary antibodies: PPARγ, C/EBPα, leptin, adiponectin, p-AMPKα, p-AMPKβ, p-ACC, HMGCR, and β-actin. Membranes were next incubated with horseradish peroxidase-conjugated secondary antibodies overnight at 4 °C. Bands were visualized with enhanced chemiluminescence, and the intensities of the bands were quantified in WCIF Image J (University Health Network Research, Toronto, ON, Canada) for Windows.

### 2.7. Statistical Analysis

Values are expressed as the mean ± standard error (SE). The evaluation of statistical significance was determined by Levene’s test followed by one-way ANOVA or Turkey HSD-test with a value of *p* < 0.05 considered to be statistically significant.

## 3. Results

### 3.1. Effects of DPG on Body Weight and Food Efficiency Ratio

**Figure 2:**
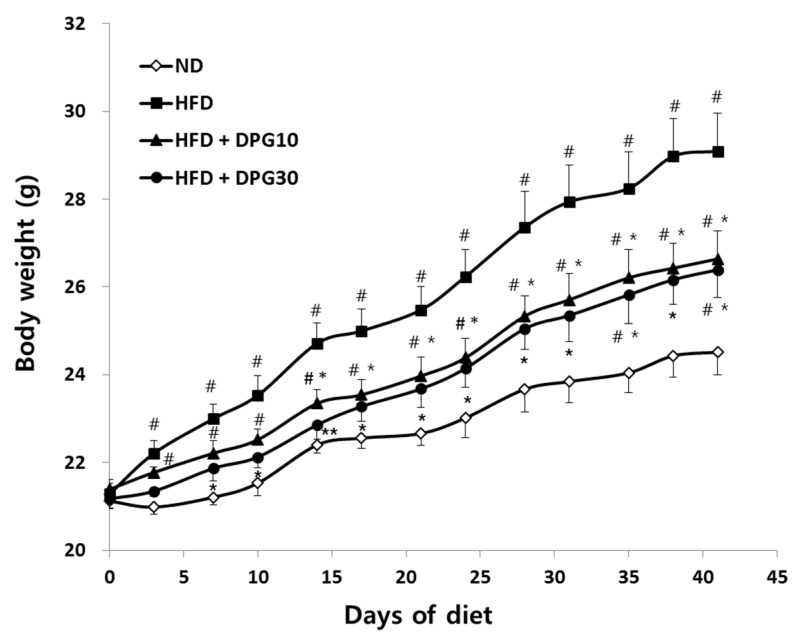
Effect of DPG10 and DPG30 on body weight in high fat-diet (HFD)-induced obese mice. Results are expressed as the mean ± SE (*n* = 12–15). ^#^ *p* < 0.05 compared with ND (normal diet) group, * *p* < 0.05, ** *p* < 0.01 compared with HFD group.

The effect of DPG on obesity was investigated using HFD-induced male C58BL/6J mice. DPG was administered to HFD-induced obese mice at doses of 10 mg/kg (DPG10) and 30 mg/kg (DPG30), respectively, for 6 weeks. In our study, the body weights was increased in the HFD group, whereas the body weight of HFD + DPG10 and HFD + DPG30 groups were significantly lower compared to HFD groups (Figure 2). On day 42, at the end of the experiment, the body-weight gains of the ND and HFD group were 3.39 g and 7.81 g, respectively. However, the body weight gains of HFD + DPG10 and HFD + DPG30-treated group were significantly lower compared to the HFD group as 5.25 g and 5.13 g, respectively (Table 1).

The food efficiency ratio (FER) of HFD was much higher compared to that of ND group. The FERs of the HFD + DPG10 and HFD + DPG30 groups were also significantly lower compared to the HFD group with no differences in food intake (Table 1).

**Table 1:** Effect of DPG on body weight gain, food intake and FER.

Group	Body Weight Gain (g/Mice/6 Weeks)	Food Intake (g/Day/Mice)	FER ^1^
ND	3.39 ± 0.48	3.49 ± 0.03	0.023 ± 0.003
HFD	7.81 ± 0.98 ^##^	2.71 ± 0.03 ^##^	0.069 ± 0.008 ^##^
HFD + DPG10	5.25 ± 0.65 ^#,^*	2.73 ± 0.04 ^##^	0.046 ± 0.005 ^##,^*
HFD + DPG30	5.13 ± 0.50 ^# *^	2.74 ± 0.09 ^##^	0.044 ± 0.004 ^##,^*

DPG: 6,8-Diprenylgenistein; FER: the food efficiency ratio; ND: normal diet; HFD: high fat diet. ^1^ Food efficiency ratio was calculated as the body weight gain/food intake. ^#^ *p* < 0.05, ^##^ *p* < 0.01 compared with ND group, * *p* < 0.05 compared with HFD group.

### 3.2. Effect of DPG on Fat Accumulation

**Figure 3:**
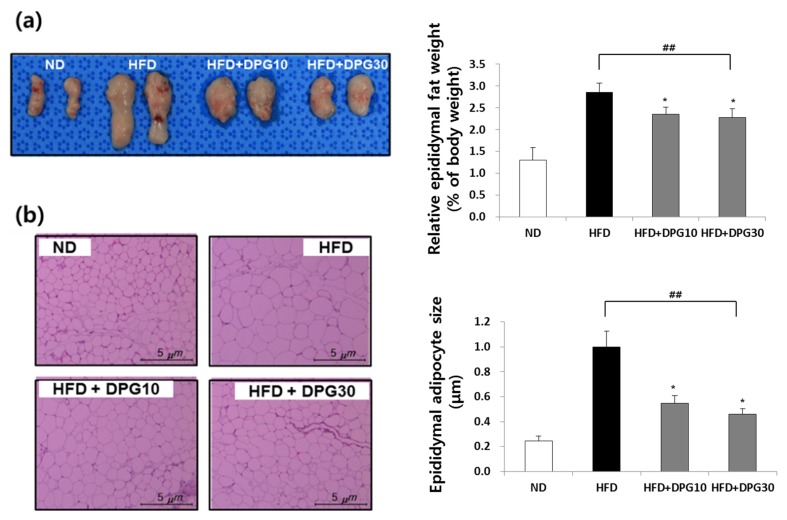
Effect of DPG10 and DPG30 on weight and size of adipocyte tissues in HFD-induced obese mice. (**a**) Epididymal adipose tissue; (**b**) sections of epididymal adipose tissue stained with hematoxylin and eosin (H&E). ^##^ *p* < 0.01 compared with ND group, * *p* < 0.05 compared with HFD group.

**Figure 4:**
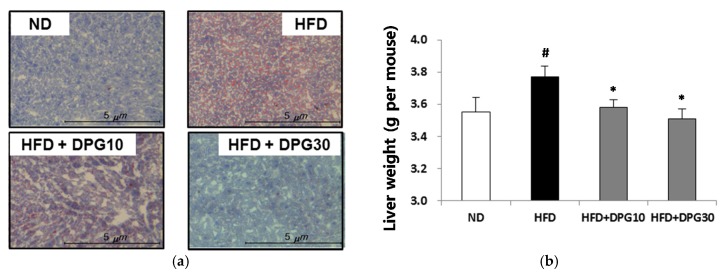
Effect of DPG10 and DPG30 on fat accumulation in liver and liver weight in HFD-induced obese mice. (**a**) Liver sections stained with Oil Red O; (**b**) liver weight per mouse. ^#^ *p* < 0.05 compared with ND group, * *p* < 0.05 compared with HFD group.

The effect of DPG on fat accumulation was examined. The relative ratio of epididymal fat per body weight was significantly higher in the HFD group compared to the ND group. The amount of epididymal fat was significantly lower in the HFD + DPG10 and HFD + DPG30 groups (Figure 3a). The epididymal adipocyte size in HFD group was markedly enlarged compared to that in ND groups. However, adipocyte sizes of HFD + DPG10 and HFD + DPG30 groups were significantly smaller than those of HFD group (Figure 3b). Fat accumulation in liver was increased in HFD group, whereas decreased in HFD + DPG10 and HFD + DPG30 groups (Figure 4a). In addition, the increase of liver weight in HFD group was also significantly reduced in HFD + DPG10 and HFD + DPG30 groups (Figure 4b).

### 3.3. Effect of DPG on Serum Lipid Profiles

Regular high fat and high cholesterol diet leads to the changes in enzyme and lipid profiles. The HFD caused elevation of the serum levels of ALT and AST which were lower in the HFD + DPG10 and HFD + DPG30 groups. Administration of DPG also significantly reduced the levels of lipid metabolic parameters including LDL, HDL and total cholesterol, and triglyceride. In particular, the HFD-induced increase of triglyceride was markedly decreased in the DPG-treated groups (Table 2).

**Table 2:** Effect of DPG on biochemical parameters of serum in HFD-induced obese mice.

Group	ND	HFD	HFD + DPG10	HFD + DPG30
AST ^1^	46.54 ± 5.55	58.85 ± 8.83	53.22 ± 5.21	50.48 ± 4.02 **
ALT ^1^	20.13 ± 2.85	24.25 ± 3.35	21.18 ± 2.14 *	20.54 ± 2.98 **
Total cholesterol ^2^	101.95 ± 10.46	151.25 ± 14.02	135.78 ± 13.44 *	130.15 ± 15.20 **
HDL cholesterol ^2^	62.61 ± 7.41	83.03 ± 6.63	76.19 ± 6.75 *	75.75 ± 6.87 *
LDL cholesterol ^2^	4.60 ± 0.82	8.17 ± 1.31	6.77 ± 1.41 *	6.45 ± 1.38 *
Triglyceride ^2^	30.71 ± 10.13	50.55 ± 13.71	35.19 ± 10.65 *	33.72 ± 7.48 **

AST: aspartate transaminase; ALT: alanine transaminase. ^1^ IU/L, ^2^ mg/dL. * *p* < 0.05, ** *p* <0.01 compared with HFD group.

### 3.4. Effect of DPG on Adipogenesis in Adipose Tissue

**Figure 5:**
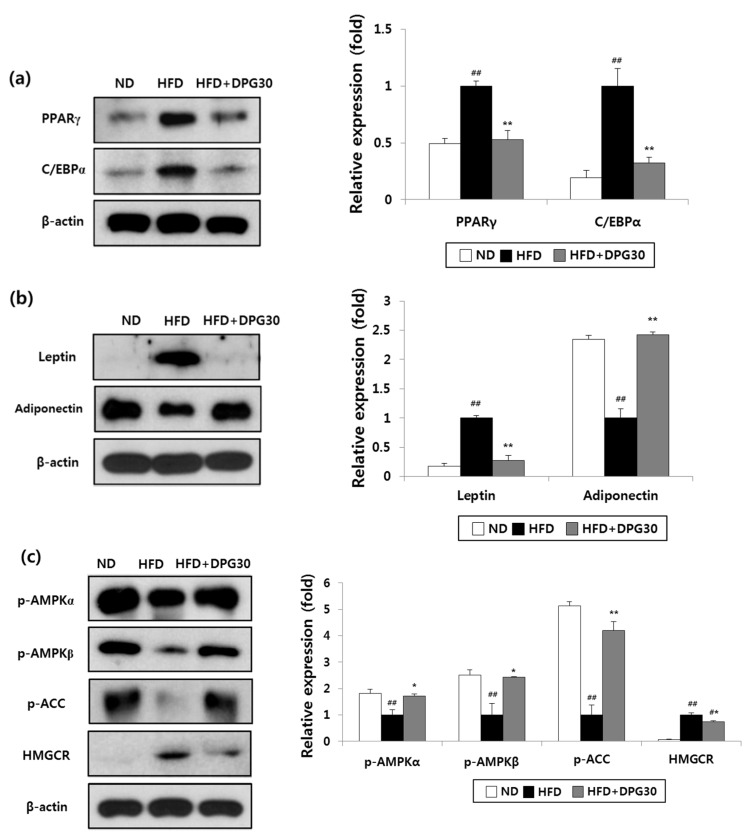
Effect of DPG on the expression of adipogenesis-related proteins in adipose tissue. (**a**) PPARγ and C/EBP α; (**b**) leptin and adiponectin; (**c**) p-AMPKα, p-AMPKβ, p-ACC, and HMBCR. Values are expressed as mean ± SE. ^#^ *p* < 0.05, ^##^ *p* < 0.01 compared with ND group, * *p* < 0.05, ** *p* < 0.01 compared with HFD group.

Adipogenesis includes increase in expression transcription factors such as PPARγ and C/EBPα [4,21]. As shown in Figure 5, the expression of PPARγ and C/EBPα in epididymal adipose tissue of HFD group was elevated up to 2-fold and 5-fold, respectively, compared to ND group. DPG markedly suppressed the HFD-induced elevation in the expression of PPARγ and C/EBPα as comparable to ND group (Figure 5a).

Adipose tissue secretes adipokines such as leptin and adiponectin. HFD cause differential effects on these adipokines [22,23]. The leptin expression was increased in HFD group, whereas adiponection expression was decreased in HFD group. DPG reversed the HFD-induced increase in leptin expression and decrease in adiponection expression (Figure 5b).

### 3.5. Effect of DPG on AMPK Pathway

AMPK is a heterotrimer consisting of a catalytic subunit (α) and 2 regulatory subunits (β and γ). AMPK, a regulator of energy homeostasis, plays a major role in lipid metabolism. AMPK activation via phosphorylation promotes phosphorylation and inactivation of acetyl CoA carboxylase (ACC), which results in reduced biosynthesis of fatty acids and stimulation of fatty acid oxidation [6,7]. As expected, phosphorylation of AMPK and ACC was reduced in HFD group and DPG promoted phorphorylation of AMPK and ACC (Figure 5c).

HMG-CoA reductase (HMGCR) is another key enzyme that control cholesterol synthesis [24]. The expression of HMGCR was increased in epididymal adipose tissue of HFD groups and was lower in HFD + DPG groups compared to that of HFD controls (Figure 5c).

## 4. Discussion

Due to the harmful effect of obesity on human health, investigations for anti-obesity therapeutics have been actively conducted in many fields. Especially, food and food ingredients are considered as good targets for anti-obesity agents to prevent obesity and obesity-associated disorders [4,25,26,27,28]. In our previous study, we suggested anti-obesity activity of *C. tricuspidata* fruits and its major isoflavonoid, DPG [18]. In this study, we investigated the anti-obesity potential of DPG in HFD-induced obese mice.

Diet-induced obesity in rodents has been used as an animal model to investigate environmental effect. Rodents fed HFD become obese and show distinctive symptoms such as increase of adipose tissue, disturbance of lipid metabolism, hyperinsulinemia and fatty liver, which are typically associated with human obesity [29]. In our present study, HFD-induced obesity was clearly confirmed by many factors such as increase of body weight, adipocyte size, fat accumulation and liver weight, and disturbance of lipid metabolism. However, oral administration of DPG, at doses of 10 and 30 mg/kg, dramatically improved HFD-induced obesity in many parameters.

First, the body weight in HFD + DPG groups were significantly lower than HFD groups (Figure 2). Food intake was higher in the ND group than in HFD groups, but did not significantly differ among the HFD, HFD + DPG10 and HFD + DPG30 groups. However, FERs of HFD + DPG10 and HFD + DPG30 groups were significantly reduced compared to HFD group (Table 1). Therefore, decrease of body weight in HFD + DPG10 and HFD +DPG30 group was achieved partially by decrease of FER not by loss of appetite. Consistent with FER, DPG inhibits pancreatic lipase, which plays key roles in fat digestion [18]. Therefore, DPG inhibited fat absorption by pancreatic lipase inhibition without any effect on appetite, which leaded the reduction of body weight gain.

Increase of fat accumulation and abnormal lipid metabolism were also observed in HFD group, which was improved in HFD + DPG10 and HFD + DPG30 groups. Especially, the epididymal fat weight and adipocyte size were greatly increased in HFD groups, which were reduced in HFD + DPG10 and HFD + DPG30 (Figure 3a,b). HFD-induced increase of the liver weight and fat accumulation in liver also improved in HFD + DPG10 and HFD + DPG30 groups (Figure 4a,b). Therefore, DPG inhibited fat accumulation into liver, which resulted in decrease of liver weight. In addition, biochemical parameters related to lipid metabolism, such as HDL, LDL, total cholesterol and triglyceride were also recovered in HFD + DPG10 and HFD + DPG30 (Table 2). Taken together, DPG mainly act on inhibition of fat accumulation, which further resulted in decrease in liver weight and lipid profiles in blood. Therefore, DPG might be effective in liver dysfunction induced by HFD, which was supported by the improvement of ALT and AST parameters in HFD + DPG10 and HFD + DPG30 groups.

Excessively absorbed fat is accumulated as adipose tissue through adipocyte differentiation. Adipogenic differentiation is a well-organized process tightly regulated by sequential activation of many transcriptional factors. PPARγ and C/EBPα play pivotal roles in adipogenesis by regulating gene expression for fat accumulation [30,31]. Adipogenesis is also regulated by leptin and adiponection, specific hormones secreted from adipose tissue [32,33]. Leptin amount is proportionally correlated with obesity, while adiponection amount is inversely related to obesity [29]. DPG effectively reduced the expression of PPARγ and C/EBPα, which were increased by HFD (Figure 5a). In addition, DPG significantly reduced leptin expression increased by HFD and restored the adiponection expression reduced by HFD (Figure 5b). Therefore, DPG efficiently reduced lipogenic genes by regulation of transcription factors and hormones, eventually leading to the suppression of lipogenesis.

To better understand the signal pathway for lipogenesis, effect of DPG on AMPK signaling was investigated. AMPK acts as an energy sensor and maintain energy homeostasis [34]. AMPK regulates fatty acid oxidation and cholesterol synthesis via ACC and HMGCR, thus has become an attractive therapeutic target in the treatment of metabolic disorders including obesity [35]. Consistent with reduced parameters of fat in epididymal, liver and serum, the levels of phosphorylation of AMPKs and ACC were higher in HFD + DPG group compared to those of HFD group. DPG also reduced expression of HMGCR (Figure 5c). In addition, DPG is a pancreatic lipase inhibitor [18]. Taken together, it is persuasive that DPG inhibited fat absorption by the inhibition of pancreatic lipase, and reduced lipogenesis via AMPK activation and followed by fatty acid oxidation and inhibition of cholesterol synthesis, which might contribute the improvement of metabolic parameters and eventually leading to the anti-obesity effect *in vivo*.

DPG is 6,8-diprenylgenistein, which means that the chemical structure of DPG is similar to genistein except for two additional prenyl moieties. Genestein, is also reported to be efficient for metabolic diseases including obesity [36]. However, DPG showed stronger inhibition on pancreatic lipase than genistein in our assay system [18], which suggested the importance of prenyl moieties of DPG. HPLC analysis showed the high content of DPG in *C. tricuspidata* fruits (Figure 1b). The extract of *C. tricuspidata* fruits contains as much as 5.4% of DPG when extracted with 70% ethanol [18]. Our present study shows that administration of 10 mg/kg is sufficient for maximum efficacy of DPG in HFD-induced obesity. Therefore, daily intake of 10–15 g *C. tricuspidata* fruits corresponds to 10 mg/kg DPG for man, which will be beneficial in regulation of obesity. In addition, *C.* *tricuspidata* fruits also contain diverse isoflavonoids including DPG and genistein [16,18]. Therefore, we suggest that DPG and *C. tricuspidata* fruits might be beneficial for the regulation of obesity, especially obesity resulting from high fat intake.

## 5. Conclusions

DPG, a major isoflavonoid of *C. tricuspidata* fruits improved many parameters of HFD-induced obesity. In particular, DPG significantly reduced epididymal fat and the serum triglyceride content, which had increased due to the HFD. Administration of DPG also improved liver dysfunction as suggested by reduced fat accumulation and the levels of ALT and AST increased by HFD. Further study suggests that DPG efficiently reduces lipogenic genes by regulation of transcription factors and hormones, and regulates ACC and HMGCR by AMPK activation. Taken together, DPG are beneficial for the regulation of obesity, especially obesity resulting from high fat intake.

## Supplementary Materials

The following are available online at http://www.mdpi.com/2072-6643/7/12/5544/s1, Table S1: Composition of experimental diets.
