# Supplementary material for: Anti-Obesity Effect of 6,8-Diprenylgenistein, an Isoflavonoid of Cudrania tricuspidata Fruits in High-Fat Diet-Induced Obese Mice
Source: Nutrients. 2015 Dec 15;7(12):10480–90. doi: 10.3390/nu7125544 (PMC4690096; doi:10.3390/nu7125544)
Supplement: Supplementary file 1 [file nutrients-07-05544-s001.docx]

**Supplementary Materials: Anti-Obesity Effect of 6,8-Diprenylgenistein, an Isoflavonoid of Cudrania tricuspidata Fruits in High-Fat Diet-Induced Obese Mice**

Yang Hee Jo, Kyeong-Mi Choi, Qing Liu, Seon Beom Kim, Hyeong-Jin Ji, Myounghwan Kim, Sang-Kyung Shin, Seon-Gil Do, Eunju Shin, Gayoung Jung, Hwan-Soo Yoo, Bang Yeon Hwang and Mi Kyeong Lee

**Table S1.** Composition of experimental diets.

| **Unit: g%** | **ND** | **HFD** |
| --- | --- | --- |
| *Formula* |  |  |
| Protein | 19.2 | 24 |
| Carbohydrate | 67.3 | 41 |
| Fat | 4.3 | 24 |
| *Ingredient* |  |  |
| Casein | 19 | 23.3 |
| l-Cystein | 0.3 | 0.3 |
| Corn starch | 29.9 | 8.5 |
| Maltodextrin | 3.3 | 11.7 |
| Sucrose | 33.2 | 20.1 |
| Cellulose | 4.7 | 5.8 |
| Soybean oil | 2.4 | 2.9 |
| Lard | 0 | 20.7 |
| Cholesterol | 0 | 0.5 |
| Mineral mixture | 0.9 | 1.2 |
| Dicalcium phosphate | 1.2 | 1.5 |
| Calcium carbonate | 0.5 | 0.6 |
| Potassium citrate | 1.6 | 1.9 |
| Vitamin mixture | 0.9 | 1.2 |
| Choline bitartrate | 0.2 | 0.2 |

ND: normal diet; HFD: high fat-diet.
